# Bilateral Orbital Plasmacytomas With Orbital Compartment Syndrome

**DOI:** 10.7759/cureus.26269

**Published:** 2022-06-23

**Authors:** Rachel E Pyon, Grace C Wang, Yan Chu, Sunil Tulpule

**Affiliations:** 1 Department of Internal Medicine, Saint Louis University School of Medicine, Saint Louis, USA; 2 Department of Hospital Medicine, John Cochran VA Medical Center, Saint Louis, USA; 3 Department of Hematology and Oncology, Banner MD Anderson Cancer Center, Phoenix, USA

**Keywords:** extramedullary tumors, plasma cell neoplasm, orbital compartment syndrome, plasmacytoma, multiple myeloma

## Abstract

Orbital plasmacytomas are uncommon soft-tissue plasma cell neoplasms that are associated with a monoclonal or myeloma protein. There are four types of plasma cell neoplasms: multiple myeloma (MM), monoclonal gammopathy of undetermined significance (MGUS), amyloidosis, and plasmacytoma. Plasmacytomas may be classified as medullary, occurring only within the bone, or extramedullary, occurring in soft tissues. Orbital plasmacytomas are extramedullary manifestations associated with MM and they may present with signs and symptoms such as unilateral proptosis, conjunctival injection, ocular pain, diplopia, and vision changes. The diagnosis of orbital plasmacytomas is based on tissue biopsy and histologic and immunohistochemical confirmation of a homogenous infiltrate of monoclonal plasma cells.

In this report, we present a case of a 60-year-old female patient with a prior diagnosis of MM and new-onset bilateral orbital plasmacytomas following an autologous peripheral blood stem cell transplant; her condition improved significantly following treatment with dexamethasone, cisplatin, doxorubicin, cyclophosphamide, and etoposide along with palliative radiation therapy (RT) of 2000 cGy in 10 fractions to the orbits. Unfortunately, three months later, she had progression of extramedullary disease with parotid gland involvement. She had multiple complicated hospitalizations and eventually expired. As patients with orbital plasmacytomas classically have lower remission and survival rates compared to those with extramedullary plasmacytomas involving other locations, they must be considered high-risk patients who require a multidisciplinary approach for early diagnosis and timely treatment in order to prevent disease progression and to alleviate symptoms related to the disease.

## Introduction

Orbital plasmacytomas are rare soft-tissue plasma cell neoplasms. Plasma cell neoplasms are associated with a monoclonal or myeloma protein. The four different types of plasma cell neoplasms are as follows: multiple myeloma (MM), monoclonal gammopathy of undetermined significance (MGUS), amyloidosis, and plasmacytoma [1,2]. Plasmacytomas can be divided into two categories: medullary (found in the bone) and extramedullary (found in soft tissues). Medullary plasmacytomas account for about 2-5% of all plasma cell neoplasms, and extramedullary plasmacytomas make up approximately 3% of all plasma cell neoplasms [3].

Plasmacytomas are associated with MM, a plasma cell neoplasm that is most often confined to the bone marrow and presents with symptoms such as bone pain, infection, hypercalcemia, anemia, and renal involvement [4]. Extramedullary plasmacytomas have been previously reported to be present at diagnosis in about 7% of MM cases [5]. Orbital plasmacytomas are uncommon types of extramedullary plasmacytoma that most often present with signs and symptoms of unilateral proptosis (81%), conjunctival injection, ocular pain, diplopia, and vision changes [6]. The diagnosis is based on tissue biopsy and histologic and immunohistochemical confirmation of a homogeneous infiltrate of monoclonal plasma cells, while CT or MRI is required to evaluate the extent of the lesion(s) [1].

In this report. we discuss a case of a patient with a prior diagnosis of MM and newly presenting bilateral orbital plasmacytomas following an autologous peripheral blood stem cell transplant.

## Case presentation

**Figure 1:**
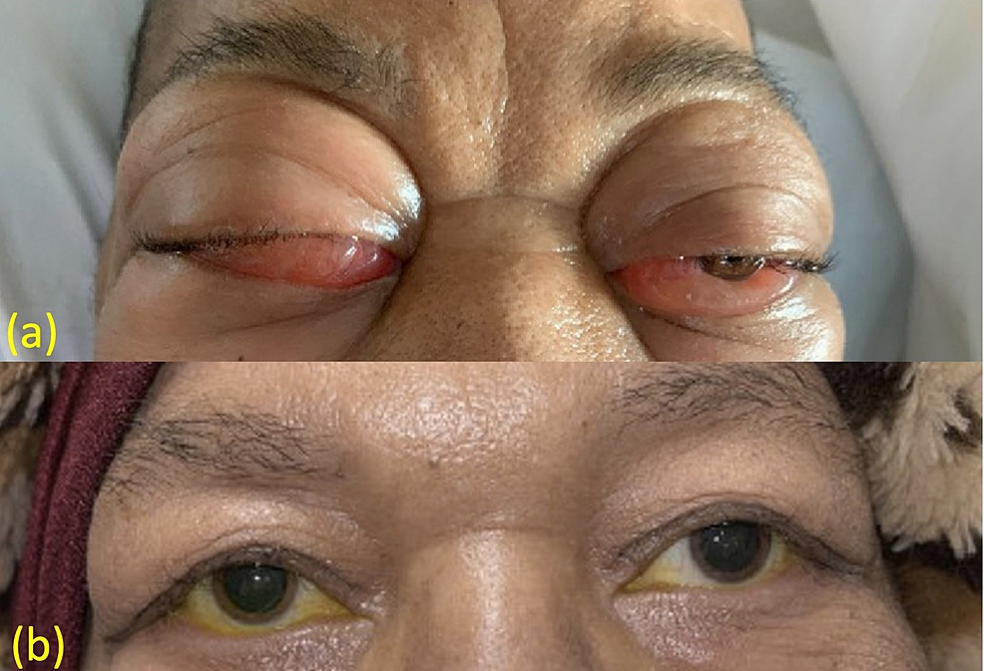
Periorbital swelling (a) On admission. (b) Two months after the completion of radiation therapy, at a follow-up ophthalmology appointment

A 60-year-old female with Revised International Staging System (R-ISS) stage II penta-refractory IgA kappa MM post autologous peripheral blood hematopoietic stem cell transplant was admitted for progressive periorbital swelling (Figure 1A) over the course of two months. The patient had initially been diagnosed with MM about two years prior to this presentation. One month after the diagnosis, the patient had undergone four cycles of an initial treatment regimen of Kyprolis, Revlimid, and Decadron, with a preconditioning regimen consisting of Cytoxan and melphalan. The patient's first report of orbital involvement had occurred about eight months following her initial diagnosis when a brain MRI had further shown multiple intraorbital masses. The patient had not been restaged at the time of the initial demonstration of bilateral orbital involvement. She received five different lines of treatment overall, including completion of two cycles of the recently FDA-approved drug for penta-refractory disease, selinexor, prior to her death. She was still receiving this treatment until her latest described situation as reported in this article, which was about 14 months since her first date of diagnosis of MM. Her last aggressive relapse had been approximately six months prior to this presentation, where imaging had shown a worsening chest wall mass, breast mass, and new orbital lesions.

**Figure 2:**
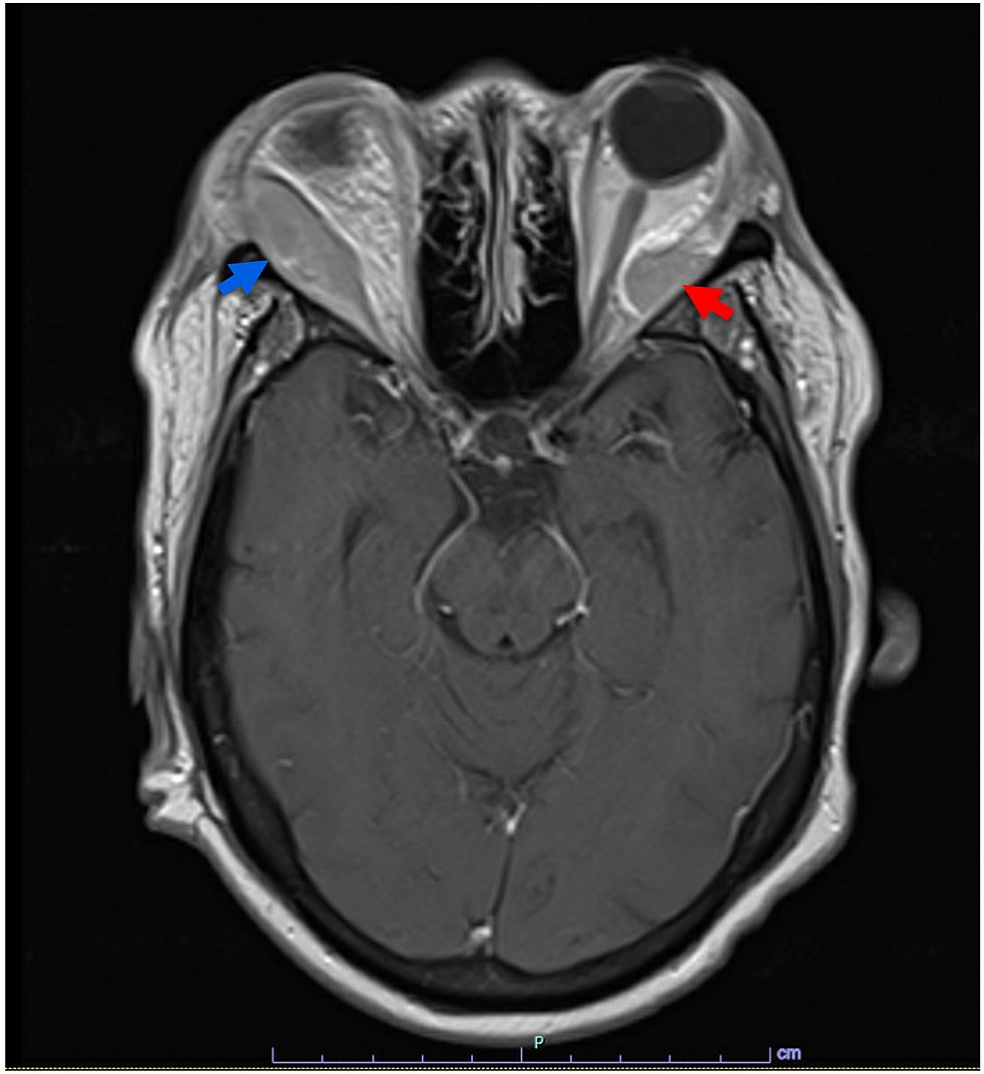
MRI brain on admission The right orbital mass (blue arrow) exerted a local mass effect on the lateral rectus muscle and the posterior lateral globe. The left orbital mass (red arrow) exerted a mild mass effect on the lateral rectus muscle MRI: magnetic resonance imaging

Under the care of her ophthalmologist, she was using loteprednol ophthalmic drops and had completed a four-day course of dexamethasone the week prior. She had significant bilateral eye pain, inability to open her right eyelid, and frontal headache, which she rated as 10/10 in severity. MRI of the brain with and without contrast (Figure 2) showed recurrence of her previous bilateral orbital masses. The right orbital mass (blue arrow) exerted a local mass effect on the lateral rectus muscle and the posterior lateral globe. The left orbital mass (red arrow) exerted a mild mass effect on the lateral rectus muscle. On admission, the patient’s visual acuity was 20/70 OD and 20/60 OS, which had changed from 20/50 OD and 20/40 OS one week prior. Intraocular pressure of her right eye was measured to be 35 mmHg (normal range: 12-22 mmHg), and intraocular pressure of her left eye was measured to be 27 mmHg. Despite 30 minutes of multiple rounds of intraocular pressure-lowering ophthalmic solution administered at the bedside, the lowest intraocular pressures recorded were 30-33 mmHg in the right eye and 23-25 mmHg in the left eye. Loteprednol drops were discontinued, one dose of intraocular acetazolamide was given, and the patient was trialed on an aggressive intraocular pressure-lowering regimen of timolol, dorzolamide, and latanoprost drops for 24 hours and initiated on high-dose Solu-Medrol (1 g intravenously daily for three days). The patient did not experience any improvement in vision or eye pain, and radiation oncology was consulted. She was treated emergently with dexamethasone, cisplatin, doxorubicin, cyclophosphamide, and etoposide along with palliative radiation therapy (RT) of 2000 cGy in 10 fractions to the orbits. She had a significant reduction in the bilateral periorbital swelling and improvement in her bilateral eye pain (Figure 1B) over the 10-day course of RT. Three months later, she had progression of extramedullary disease with parotid gland involvement. She had multiple complicated hospitalizations and eventually expired.

## Discussion

It is essential to distinguish solitary plasmacytoma from MM, as their prognoses and treatments differ. Extramedullary plasmacytomas are highly radiosensitive with high success rates of long-term stability and cure. While RT is the standard treatment for solitary plasmacytoma, its role is much less defined in MM with extramedullary disease [6]. The patient in this case received systemic chemotherapy in addition to palliative RT given her disease progression. Furthermore, it is also crucial to distinguish between solitary extramedullary plasmacytoma and plasmacytoma as a late manifestation of MM following many treatment lines, as the former has a favorable prognosis and the latter represents a rapidly developing aggressive tumor with a poorer prognosis.

The frequency of extramedullary plasmacytoma as a manifestation of MM is on the rise nowadays, particularly following the use of multisystemic treatment modalities. Particularly, the anti-CD38 monoclonal antibodies appear to favor the manifestation of multiple extranodal plasmacytomas [7]. Orbital plasmacytoma can be the presenting symptom of undiagnosed MM, or of uncontrolled and recurrent disease, such as in this case. The vast majority of these patients have unilateral involvement, with proptosis being the most common finding [4]. This case is an atypical presentation due to the bilateral involvement (12% of cases). The progression is most often insidious, with a duration of five months on average from symptom onset to presentation. Patients with orbital plasmacytoma have lower remission and survival rates compared to those with extramedullary plasmacytomas involving other locations (28 months vs. 8.3 years) [5]. Data in the literature suggest that extramedullary involvement as a late sequella of MM negatively affects survival among these patients [6].

## Conclusions

This report presented a case of a patient with a prior diagnosis of MM and bilateral orbital plasmacytomas, with significant improvement in ocular symptoms after palliative RT. Documented survival rates of patients with extramedullary plasmacytomas are low, regardless of the tissue in which the tumors present. These high-risk patients, including those with orbital plasmacytoma, require a multidisciplinary approach for early diagnosis and timely treatment to achieve symptom relief and prevent disease progression. Further studies aimed at evaluating and defining the most optimal management plan for extramedullary MM are required.
